# A dataset of housing market and self-attitudes towards housing location choices in Alexandria, Egypt

**DOI:** 10.1016/j.dib.2017.02.052

**Published:** 2017-03-09

**Authors:** Mohamed R. Ibrahim

**Affiliations:** Department of Urban Development, Technische Universität Berlin-Campus El Gouna, El Gouna, Egypt

**Keywords:** Housing market in Alexandria, Egypt, Factors of residential location choice, Housing demand preferences, Travel behavior

## Abstract

A survey, of sample size 224, is designed to include the different related-factors to housing location choice, such as; socioeconomic factors, housing characteristics, travel behavior, current self-selection factors, housing demand and future location preferences. It comprises 16 questions, categorized into three different sections; socioeconomic (5 Questions), current dwelling unit characteristics (7 Questions), and housing demand characteristics (4 Questions). The first part, socioeconomic, covers the basic information about the respondent, such as; age, gender, marital status, employment, and car ownership. While the second part, current dwelling unit characteristics, covers different aspect concerning the residential unit typology, financial aspects, and travel behavior of the respondent. It includes the tenure types of the residential unit, estimation of the unit price (in the case of ownership or renting), housing typologies, the main reason for choosing the unit, in case of working, the modes of travel to work, and time to reach it, residential mobility in the last decade, and the ownership of any other residential units. The last part, housing demand characteristics, covers the size of the demand for a residential unit, preference in living in a certain area and the reason to choose it, and the preference of residential unit׳s tenure. This survey is a representative sample for the population in Alexandria, Egypt. The data in this article is represented in: How do people select their residential locations in Egypt? The case of Alexandria; JCIT1757.

**Specifications Table**

**Table:** 

Subject area	*Urban Studies*
More specific subject area	*Residential location choices, housing market conditions*
Type of data	*Tables, and figures*
How data was acquired	*Conducting a questionnaire*
Data format	*Raw, Analyzed*
Experimental factors	*Highlighting the individual preferences towards housing location choices based on the current housing market conditions.*
Experimental features	*The relation between housing self-selection process, affordability and market conditions.*
Data source location	*Alexandria, Egypt*
Data accessibility	*Data is available with a published article*[4].

**Value of the data**

• This dataset provides a first-hand survey that explores the topic of residential location choices in the context of Egypt, specifically in case study of Alexandria.
• It provides census update to the city of Alexandria in term of housing demand and characteristics.
• This data can open the door for different types of research that tackle housing characteristics socioeconomic factors and travel behavior in developing countries.

## 1 Data

It is a raw data collected by a survey of a sample size 224. It consists of 16 questions related to different factors that affect the self-selection of housing location choices such as: socioeconomic factors, unit characteristics, travel behavior, in addition to demand size and the future expectation and the previous residential mobility.

## 2 Experimental design, materials and methods

The sample size is calculated based on survey sample size calculator. A sample of 224 respondents has been estimated based on four attributes. First, the population size of Alexandria estimated as 4,546,231 in 2014. Second, a confidence level of 95 percent is conducted. Third, confidence interval, or precision, estimated to be 6.55, and last, an average distribution of the responses of 50 percent to the different questions of the survey [2], [3], [6], [8].

The stratified random sampling method was conducted to ensure the representation of the wide spectrum of the population [1, 5]. Three different stages are used to sub-group the population. Stage one subdivided the sample according to the weight of the inhabitants of each district of Alexandria. Similarly, stage two subdivided the sample of each district in proportion to the weight of population in each selected, most populated, Sheykhat.^1^ These two steps ensure the representation of sample according to the geographical distribution of the population. In stage three, the stratified random sample subdivided the population into strata according to four variables, age, gender, car ownership, and estimated housing price to ensure the representation of the different socioeconomic groups [7]. For instance, females represent 48.9 percent of the total population in Egypt. 60.2 percent of the population is under the age of 30 years while 27.7 percent is at the age of 21–35, 11.6 percent is between the age 36–45, last, 20.1 percent is either 46 years or above [9]. On the other hand, it was essential for this research to consider the representation of the strata according to car ownership per household and the estimated owning/rental price of dwellings to include various socioeconomic classes.

## Appendix A Supplementary material

.

Supplementary material. PDF file for the questionnaire illustrating the different tackled questions. Excel sheet for the dataset coded based on the orders of the different categories of each question in the survey template, in addition to SPSS format file.

## Footnotes

1 Literally provinces, plural of Sheyakha, the smallest planning unit in the Egyptian planning system of cities with defined administrative boundaries.
