# Supplementary material for: A dataset of housing market and self-attitudes towards housing location choices in Alexandria, Egypt
Source: Data Brief. 2017 Mar 9;11:543–5. doi: 10.1016/j.dib.2017.02.052 (PMC5357685; doi:10.1016/j.dib.2017.02.052)
Supplement: Supplementary file 1 — Supplementary material [file mmc1.docx]

I, Mohamed R. Ibrahim, justify that there is no conflict of interest.

N.B. I can’t find any official form to fill in.
