# Supplementary material for: A dataset of housing market and self-attitudes towards housing location choices in Alexandria, Egypt
Source: Data Brief. 2017 Mar 9;11:543–5. doi: 10.1016/j.dib.2017.02.052 (PMC5357685; doi:10.1016/j.dib.2017.02.052)
Supplement: Supplementary file 4 — Supplementary material. PDF file for the questionnaire illustrating the different tackled questions. Excel sheet for the dataset coded based on the orders of the different categories of each question in the survey template, in addition to SPSS format file. [file mmc4.pdf]

RESIDENTIAL LOCATION CHOICE SURVEY

PART I: SOCIO-ECONOMICS

1. AGE: ○ Between 21-35 ○ Between 36-45 ○ 46, or above

2.GENDER: ○ Male ○ Female

3.MARITAL STATUS : ○ Single ○ In a relationship ○ Married ○ Divorced ○ Widow

4.EMPLOYMENT: ○ Full time job ○ Part-time job ○ Work at home ○ Searching for a job ○ Retired

5. I /MY FAMILY OWN: ○ No car ○ One car ○Two cars ○ More than two cars

PART II: CURRENT DWELLING UNIT CHARACTERISTICS

6.WHAT TYPE OF TENURE IS YOUR HOUSE?

○ Owned ○ Old rent lease ○ New rent lease

-In case of Ownership, what would be the actual price now of your unit in Real Estate market?

○ Less than EGP 99,999 ○ Between EGP 100,000 - 199,999 ○ Between EGP 200,000 -299,999

○ Between EGP 300,000 - 399,999 ○ EGP 400,000, or above

-In case of Renting, you pay

○ Less than 1/3 of your income ○ Between 1/3 to 1/2 of your income

○ More than 1/2 of your income

7.WHAT TYPE OF HOUSE DO YOU LIVE IN? - Average Area (m2): ....

○ Private apartment ○ Public housing ○ Corporative housing

○ Self-built house ○ Others, please specify .....

8.WHY DID YOU CHOOSE THE PLACE YOU ARE LIVING IN NOW ? **(Please select the most 3 relevant factors)**

Because; ○ I afford this house ○ Proximity to work

○ Family's asset ○ Availability of transportation

○ Nice Neighborhood ○ Proximity to family / relatives

○Nearby Downtown ○ Social Standing of the area

9. HOW LONG DOES IT TAKES YOU TO GO TO WORK ?

○ Less than 30 min ○ Between 30-60 min ○ Between 61-59 min

○ 90 min, or more ○ I work outside Alexandria

10. WHICH MAIN MODE OF TRANSPORTION DO YOU USE TO GO TO WORK ?

○ Walking ○ My Private Car ○ Bus or Micro-bus ○ Train or Tram line

○ Taxi ○ Ride in a friend's car ○ Transport facility offered by the company

11. HAVE YOU CHANGED YOUR HOUSE BEFORE? ○ YES ○ NO

IF **YES**, when was the last time you moved ?

○ Less than two years ○ From 2 to 10 years ○ More than 10 years

The main reason to move was: ○ Unit type /or Neighborhood ○ tenure type ○ Transports ○ Others

12. DO YOU, OR YOUR HOUSEHOLD MEMBERS OWN ANOTHER HOUSING UNIT ?

○ No ○ YES , How many? .... which it is / they are now: ○ Vacant ○ Occupied

PART III: HOUSING DEMAND CHARACTERISTICS

13. ARE YOU, OR ONE OF YOUR HOUSEHOLD'S MEMBERS SEARCHING FOR A NEW HOUSE?

○ Yes ○ No

14. IN WHICH DISTRICT WOULD YOU PREFER TO LIVE?

○ Same neighborhood ○ New Borg El Arab

○ Other old districts in Alexandria ○ Outside Alexandria, Specify...

15. THE MAIN REASONS TO CHOOSE A NEW LOCATION ARE.... **(Please select the most 3 relevant factors)**

○ Reasonable price ○ Proximity to transportation

○ Proximity to work ○ Proximity to social relations and relatives

○ Quiet neighborhood ○ Social standing of the area

○ Services and utilities ○ Security and Safety

16. WHICH TENURE TYPE WOULD YOU PREFER?

○ Ownership ○ Long-Term Rent ○ Short-Term Rent
